# Host diet mediates a negative relationship between abundance and diversity of *Drosophila* gut microbiota

**DOI:** 10.1002/ece3.4444

**Published:** 2018-08-29

**Authors:** Berra Erkosar, Erika Yashiro, Felix Zajitschek, Urban Friberg, Alexei A. Maklakov, Jan R. van der Meer, Tadeusz J. Kawecki

**Affiliations:** ^1^ Department of Ecology and Evolution University of Lausanne Lausanne Switzerland; ^2^ Department of Fundamental Microbiology University of Lausanne Lausanne Switzerland; ^3^ Department of Animal Ecology Evolutionary Biology Centre Uppsala University Uppsala Sweden; ^4^ Evolution and Ecology Research Center School of Biological, Earth and Environmental Sciences University of New South Wales Sydney NSW Australia; ^5^ IFM Biology AVIAN Behavioural Genomics and Physiology Group Linköping University Linköping Sweden

**Keywords:** diet, *Drosophila*, host–microbe interactions, intermediate productivity hypothesis, microbiota, productivity–diversity relationship

## Abstract

Nutrient supply to ecosystems has major effects on ecological diversity, but it is unclear to what degree the shape of this relationship is general versus dependent on the specific environment or community. Although the diet composition in terms of the source or proportions of different nutrient types is known to affect gut microbiota composition, the relationship between the quantity of nutrients supplied and the abundance and diversity of the intestinal microbial community remains to be elucidated. Here, we address this relationship using replicate populations of *Drosophila melanogaster* maintained over multiple generations on three diets differing in the concentration of yeast (the only source of most nutrients). While a 6.5‐fold increase in yeast concentration led to a 100‐fold increase in the total abundance of gut microbes, it caused a major decrease in their alpha diversity (by 45–60% depending on the diversity measure). This was accompanied by only minor shifts in the taxonomic affiliation of the most common operational taxonomic units (OTUs). Thus, nutrient concentration in host diet mediates a strong negative relationship between the nutrient abundance and microbial diversity in the *Drosophila* gut ecosystem.

## 1 INTRODUCTION

The availability of nutrients is a major factor affecting the diversity of ecological communities, but the shape of the relationship between nutrient supply and diversity and the degree to which it generalizes across different types of communities and ecosystems remains a subject of controversy (Chase & Myers, 2011; Huston, 2014; Smith, 2007). Patterns from plant and animal communities are variable, although a decrease in diversity in highly nutrient‐enriched communities is almost universally observed (Dickson & Foster, 2011; Fridley et al., 2012; Humbert, Dwyer, Andrey, & Arlettaz, 2016; Huston, 2014; Maskell, Smart, Bullock, Thompson, & Stevens, 2010). In contrast, multiple experimental studies found an increase in alpha diversity with nutrient supply in soil bacteria (Canfora et al., 2015; Doan et al., 2014; Kearns, Angell, Feinman, & Bowen, 2015; Zhang et al., 2012; Zhen et al., 2014; Zhong et al., 2010) and aquatic protists (Haddad et al., 2008; Johnson & Angeler, 2014; Scholes, Warren, & Beckerman, 2005); the patterns in aquatic bacteria are variable (Smith, 2007). Thus, the relationship between nutrient availability and diversity may differ between types of communities, reflecting differences in way their members compete, disperse, and evolve.

In this study, we address the relationship between nutrient supply—determined by the nutritional content of the host's diet—and diversity of the microbial community of the animal digestive system. Compared to other microbial environments such as soil, the gut is characterized by a high influx of nutrients and the involvement of the host immune system in regulation of the community (Hooper, Littman, & Macpherson, 2012), factors that may affect the responses of community structure to diet. Studying this relationship is not only interesting from the viewpoint of understanding the gut as an ecosystem, but is also important from the viewpoint of the host because the abundance, composition, and diversity of the gut microbial community affect the host's physiology (Douglas, 2015; Li et al., 2008), immunity (Kau, Ahern, Griffin, Goodman, & Gordon, 2011), behavior (Ezenwa, Gerardo, Inouye, Medina, & Xavier, 2012), and fitness (Ruokolainen, Ikonen, Makkonen, & Hanski, 2016). Multiple studies have shown that changes in host diet can have large and rapid effects on the composition of the gut microbiota in mammals (e.g., Carmody et al., 2015; David et al., 2014; Turnbaugh, Baeckhed, Fulton, & Gordon, 2008), fish (e.g., Bolnick et al., 2014), and insects (e.g., Chandler, Lang, Bhatnagar, Eisen, & Kopp, 2011; Perez‐Cobas et al., 2015). Of several studies that reported alpha diversity indices, some found considerable effects of host diet on microbial alpha diversity (Bolnick et al., 2014; Ounnas et al., 2016; Turnbaugh et al., 2008), whereas others did not, despite observing large changes in microbiota composition (David et al., 2014; Perez‐Cobas et al., 2015). However, these studies manipulated diet composition in terms of the type or source of nutrients, (e.g., fat vs. protein rich or of plant vs. animal origin) rather than over nutrient amount or concentration. Thus, the existing literature throws little light on the relationship between the quantity of nutrients and the diversity and abundance of the microbiota.

To address this gap, we studied how the total abundance and diversity of the intestinal microbiota of *Drosophila melanogaster* is affected by the concentration of yeast in the flies’ diet. Yeast was the sole source of protein and most other nutrients in our experimental diets, and it is also the key source of nutrition for *Drosophila* in their natural habitats (Powell, 1997). We analyzed the microbiota of experimental fly populations maintained for 40 months (approximately 60 fly generations) on diets containing 4%, 10%, or 27% yeast in population cages with overlapping generations, favoring the natural transmission of microbiota via consumption of feces. Our findings indicate that the nutrient supply in this system mediates a negative relationship between microbiota abundance and diversity.

## 2 MATERIALS AND METHODS

### 2.1 Fly husbandry and experimental design

The populations of *D. melanogaster* used in this study were set up in the Maklakov Lab (EBC, Uppsala University) (Zajitschek et al., 2016). They were initiated from an outbred stock originating from a wild population collected in Dahomey (now Benin) in 1970. Adults of the experimental populations were kept in population cages and fed with food dishes containing 40, 100, or 270 g of brewer's dry yeast per liter (further referred to as 4%, 10%, and 27% diets, respectively), with four replicate populations per diet. Yeast was the only source of dietary protein; the food medium in all cases also contained 50 g of sucrose, 15 g of agar, and the antifungal agents Nipagin (3 g) and propionic acid (3 g) per liter. The food dishes were replaced twice a week. To propagate the populations, every week eggs were collected from food bottles and transferred to fresh vials with the 10% food at a density of ~100 eggs per vial. These vials were incubated outside of the cages; adults developed in them were used to replenish the populations in the cages. Thus, only adults faced different dietary regimes; all larvae were raised on the standard 10% diet. The adult populations were maintained at the census size of 150 males and 150 females, which was achieved by replacing dead flies by newly emerged adults (0–36 h old) once per week. The cages were closed with a nylon mesh and haphazardly placed in an incubator with constant air flow at 25°C and 60% humidity with a 12 h:12 h light:dark cycle. No attempt was made to prevent the dispersal of microbes among cages; for example, the hands of the person exchanging the food dishes were not sterilized between handling cages. Thus, the cages are likely to have shared a pool of microbes, and any differences observed are unlikely to be due to stochastic founder effects. By the time of microbiota sampling, this experiment had been running for 40 months.

To obtain samples of gut microbiota, six males and six females were haphazardly collected from each population cage before the addition of new adults (i.e., the sampled individuals were at least one week old). Animals were anesthetized by chilling on ice and subjected to gut dissections in sterile PBS (phosphate‐buffered saline, pH 7.4) using surface sterilized tools. Dissected guts were frozen in liquid nitrogen. As a negative control for bacterial community analysis, “mock” sampling was conducted without flies but with analogous manipulations in PBS with the same dissection tools.

### 2.2 DNA extraction and 16S rRNA amplicon sequencing

The 12 dissected guts from each population were pooled for DNA extraction. Genomic DNA was extracted from dissected guts or from the same volume of the mock sample using the cetyltrimethylammonium bromide/phenol protocol (Powell, Martinson, Urban‐Mead, & Moran, 2014). Consistent with other studies of microbiota in *Drosophila* (Staubach, Baines, Kunzel, Bik, & Petrov, 2013; Wong et al., 2015), community composition was assessed based on the V1‐V2 regions of the 16S rRNA gene. These regions were amplified using the KAPA HiFi HotStart ReadyMix (Kapa Biosystems # KK2601) and primers 8‐27F: 5′‐TCGTCGGCAGCGTCAGATGTGTATAAGAGACAGAGAGTTTGATCMTGGCTCAG‐3′ and 339‐356R: 5′‐GTCTCGTGGGCTCGGAGATGTGTATAAGAGACAGTGCTGCCTCCCGTAGGAG‐3′ including adapter sequences (underlined) for the second PCR round. Three‐replicate 25 μl PCRs containing 10 ng/μl DNA and 1 μM of each primer were carried out under the following conditions: 95°C 3 min, 25 cycles of 95°C 30 s‐56°C 15 s‐72°C 30 s, followed by a final incubation at 72°C for 5 min. Products were pooled from triplicate reactions and verified for amplicon size on a Fragment Analyzer (Advanced Analytical Technologies, Inc.). In case of mock PBS sample, a volume of 2 μl was amplified in the PCR and processed in the same volumetric manner as the other samples. Libraries were prepared and sequenced at the Lausanne Genome Technology Facilities of the University of Lausanne according to the Illumina 16S Metagenomic Sequencing Library Preparation protocol.

### 2.3 OTU calling and filtering

All steps of sequence analysis were performed using the QIIME 1.8.0 bioinformatics software (Caporaso et al., 2010b). Raw 300‐bp paired‐end reads were filtered by size (minimum 100 bp overlap between paired ends) and quality (phred scores ≥ 30). Chimeric reads were eliminated using the Usearch (QIIME dependency, v6.1) algorithm (Edgar, 2010; Edgar, Haas, Clemente, Quince, & Knight, 2011). Reads were classified into operational taxonomic units (OTUs) using the open reference OTU clustering pipeline, excluding the prefiltering step and using the *uclust* method (Edgar, 2010). Reads were aligned to the Greengenes database (Desantis et al., 2006) using PyNAST (Caporaso et al., 2010a) with 97% identity threshold. Less than 1% of the reads failed to be mapped to bacteria. Singletons were discarded. Phylogenetic trees were built using FastTree 2.1.3. (Price, Dehal, & Arkin, 2010).

Taxonomies were assigned using the RDP classifier (2.12) on representative sequences of each identified OTU, using bootstrap confidence threshold of 0.95 (Wang, Garrity, Tiedje, & Cole, 2007). The flies used in this study carry the bacterial endosymbiont *Wolbachia,* which lives inside of host cells rather than being part of gut microbiota (e.g., Staubach et al., 2013). As the 16 rRNA gene of *Wolbachia* is also amplified by the universal primers used here, a majority of reads came from *Wolbachia*, reducing the sampling depth of microbiota communities (this is common in *Drosophila* microbiota analysis, e.g., (Adair, Wilson, Bost, & Douglas, 2018)). This was particularly the case in samples from the 4% yeast diet, presumably because of the low total absolute microbiota abundance. This resulted in relatively few reads attributed to microbiota in those samples (Data File 1 available on Dryad).

Putative microbiota OTUs were subjected to two further steps of filtering before the analysis of community composition. First, to filter out spurious OTUs resulting from sequencing or amplification errors, we retained OTUs that were present in at least three samples (excluding the mock sample) and were represented by at least 24 reads in total across the 12 samples. Eighty‐three OTUs passed this filtering step, 26 of which were also detected in the mock sample. In the second filtering step, the mock sample was used to exclude OTUs whose presence in the gut samples could be explained by contamination during sampling or the sequencing process, that is, whose absolute abundance in the gut samples was similar to that in the mock sample. We estimated the absolute abundance of OTU *i* in sample *j* as

$$\begin{array}{cc} & \frac{\text{number of reads of OTU}\;i\;\text{in sample}\;j}{\text{total number of 16S reads in sample}\;j}\\ & \times \;\text{concentration of 16S DNA in sample}\;j \end{array}$$

The concentration of 16S DNA in each sample was estimated in the 12 gut samples relative to the “mock” sample with qPCR, with universal primers developed for this purpose by Kesnerova et al. (2017); primer efficiency was 103%. To be retained, the estimated abundance of an OTU in at least three‐gut samples had to be at least 10‐fold higher than in the mock sample. This eliminated 17 of the 83 OTUs that passed the first filtering step, leaving 66 OTUs for the community analysis (Data File 1 on Dryad).

### 2.4 Community data and statistical analysis

To correct for the large and variable proportion of reads mapping to *Wolbachia*, the analysis of microbiota diversity among samples was performed on data rarefied to a common number of reads corresponding to the smallest sample size of 1779 (Gotelli & Colwell, 2001). Because rarefaction involves random subsampling, we performed our analyses on multiple independently rarefied data sets (1000 runs for alpha diversity and 100 for beta diversity analyses). This was performed using the “rarefy_even_depth” function in R package “Phyloseq” (Mcmurdie & Holmes, 2013).

We quantified alpha diversity with three indices: (a) Shannon's Diversity Index, which quantifies OTU diversity without taking phylogenetic relationships into account (using “estimate_richness” function in “Phyloseq”); (b) phylogenetic diversity (Cadotte et al., 2010), which quantifies the total phylogenetic tree branch length connecting species present in the community, irrespective of their abundance (using the “pez” package); and (c) abundance‐weighted phylogenetic diversity (Vellend, Cornwell, Magnuson‐Ford, & Mooers, 2011), in which the branch length is weighted by abundance of subtending OTUs (using a custom script). Following the recommendation by Louca, Doebeli, and Parfrey (2018), we did not attempt to correct for differences in the copy number of rRNA gene. To test for a relationship between these measures of alpha diversity and the host diet, we fitted linear regression with the mean diversity index values from 1000 data sets independently rarefied to the common depth of 1779 as the response and yeast concentration in the diet as the predictor variable.

To verify the robustness of our conclusions about alpha diversity to analytical details, we performed three additional variants of the above analysis: (a) treating diet as a categorical rather than continuous variable, (b) rarefying to 1334 reads (75% of the smallest sample), and (c) only retaining 20 OTUs that were most abundant on average across the samples.

Beta‐diversity analysis was based on two community distance metrics, Bray–Curtis (Bray & Curtis, 1957) and weighted UniFrac (Lozupone, Hamady, Kelley, & Knight, 2007). Both distance matrices were generated with the function “distance” and projected in nonmetric multidimensional scaling (NMDS) space using “ordinate” in “Phyloseq”. This was carried out separately for each of 100 independently rarefied data sets, resulting in 100 estimated distance matrices for each distance metric. Rather than combining these 100 matrices in one, we performed the downstream analyses on each of them separately, and report the median case and the range statistics, including *p* values. We first used permutational multivariate analysis of variance (Anderson, 2001a,b), implemented in “adonis” in “Vegan”, to test for systematic effect of host diet (i.e., yeast content, treated as a continuous variable) on community composition. Second, we tested whether variation among the replicate communities (Anderson, 2006; Anderson, Ellingsen, & Mcardle, 2006) exposed to the same host diet differed among the diet treatments. We quantified this variation using a multivariate equivalent of variance, defined as Σ*d*_*i*_^2^/(*n* – 1), where *d*_*i*_ is the Euclidean distance of the *i*th community from the treatment centroid, and the sum is taken over all *n *=* *4 replicate communities per treatment. Homogeneity of this variation across diets was tested with a permutation‐based test using “permutest.betadisperse” function in “Vegan” (Anderson, 2006).

### 2.5 Quantification of microbiota abundance

We used two complementary approaches to quantify the effect of the diet treatment on the absolute abundance of gut microbiota. First, we compared the ratio of the number of 16S microbiota reads to the number of reads attributed to *Wolbachia*. Second, we estimated the abundance of two common bacterial taxa in the *Drosophila* gut, using qPCR to quantify the amount of 16S rRNA gene relative to a host autosomal gene (*rp49*). In the absence of primers that would amplify the 16S rRNA gene off all microbiota but not *Wolbachia*, we quantified the abundance of the phylum Firmicutes and of the family Acetobacteraceae, which together accounted for between 86% and nearly 100% of reads in each sample. For Firmicutes, we used the published phylum‐specific primers: F: 5′‐TGAAACTYAAAGGAATTGACG‐3′ and R: 5′‐ACCATGCACCACCTGTC‐3′ (Bacchetti De Gregoris, Aldred, Clare, & Burgess, 2011). To quantify Acetobacteraceae, we designed custom primers based on an alignment of the 16S rRNA gene sequences of *Acetobacter aceti*,* Gluconacetobacter rheaticus*, and *Wolbachia pipientis*. The following primer regions are conserved in acetic acid bacteria but not *Wolbachia* and were chosen for qPCR: F: 5′‐GGCATGCTTAACACATGCAAG‐3′, R: 5′‐ CAGGCGACTTGCGCCTTTGAC‐3′. Primer specificity has been verified bioinformatically. To quantify *Drosophila* genome copy numbers, we used the rp49 primers F: 5′‐GACGCTTCAAGGGACAGTATCTG‐3′and R: 5′‐AAACGCGGTTCTGCATGA‐3′ (Gottar et al., 2002). The reactions were carried out with two technical replicates per sample, using 3 μM primers and the Power SYBR Green PCR Master Mix (Life Technologies, #4368702) under the following conditions: 95°C 10 min, 40 cycles of 95°C, 15 s and 60°C, 1 min. Melting curve analysis ensured amplification of a single product. Primer efficiencies (quantified using a series of five 10‐fold serial dilutions of a haphazardly picked sample) were 100% and 103%, respectively.

Each of these two approaches to quantifying absolute microbiota abundance has a limitation. First, the ratio of microbiota to *Wolbachia* reads could be confounded by potential changes in *Wolbachia* titers, which might be affected by diet (a slight increase in somatic *Wolbachia* titers on a richer diet is reported by Serbus et al. (2015)). Second, the ratio of microbiota to host genomic DNA might potentially be confounded by differences in the ploidy of gut cells (*Drosophila* enterocytes are polyploid, although no effect on diet on their ploidy has not been reported). Because the potential confounding factors are different in the two approaches, and because they use different data (reads vs. qPCR), they are complementary, and an agreement between them would not be expected if either of these confounding factors played a major role. For the analysis, ratios of bacterial taxa 16S rRNA gene to *Wolbachia* 16S rRNA and to *Drosophila* genome copy numbers were log‐transformed. To test for a relationship between diet and taxa abundance, we performed linear regression of the log‐transformed ratios on yeast concentration; additionally, we verified whether the results held if diet was treated as a categorical variable.

## 3 RESULTS

### 3.1 Microbiota abundance increases with increasing nutrient concentration

**Figure 1:**
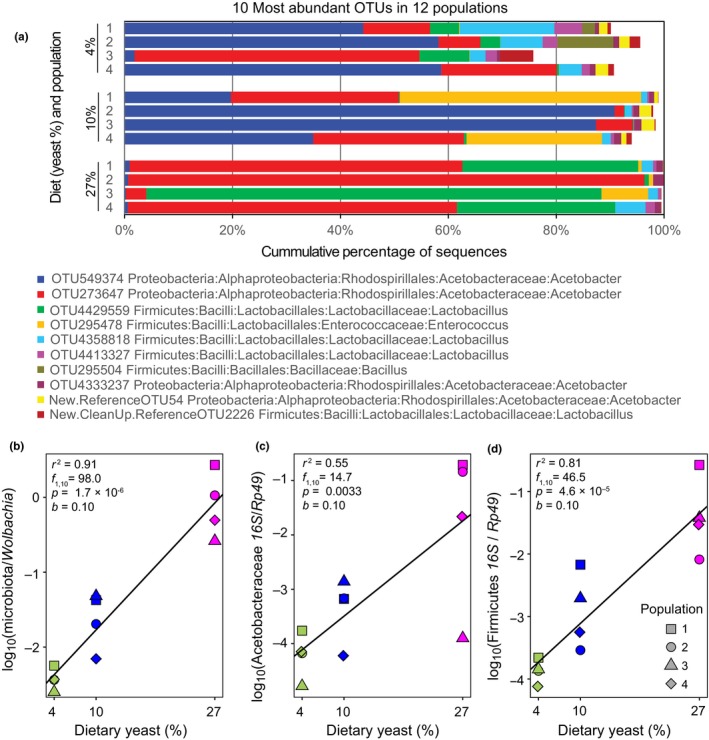
Structure of microbial gut communities and their abundances in *Drosophila* populations maintained on different diets. (a) The identities and relative abundances of 10 most abundant taxa in all populations as assigned by 16S rRNA gene amplicon sequence analysis. (b) The abundance of gut microbiota relative to the intracellular symbiont *Wolbachia*, estimated as the ratio of the 16S rRNA gene amplicon read counts. (c, d) Abundance of two dominant higher microbiota taxa, *Acetobacteraceae* (c) and *Firmicutes* (d), relative to host DNA, quantified by qPCR using taxon‐specific primers for bacteria and *Drosophila Rp49* gene primers for the host. The lines in panels b–d represent linear regression on the log scale, and the statistics refer to the slope (*b*), adjusted *R* ^2^, and the significance test of the regression. The effect of diet remains significant if it is treated as a categorical variable (*p *<* *0.0001, *p *=* *0.016, *p *=* *0.0003 in panels b, c, d, respectively; in all cases, the 27% diet is different from the 4% diet by Tukey's test). Symbol shapes identify different replicate host populations (their numbering is arbitrary, i.e., Population 1 from 4% diet is not paired with Population 1 from 10% or 27% diet)

We used 16S rRNA gene sequencing to compare the gut microbial communities of experimental populations of *D. melanogaster* maintained on three diets differing in yeast concentration (4%, 10%, and 27% yeast weight/volume). We identified a total of 66 operational taxonomic units (OTUs) that passed filtering (Data File 1 on Dryad). Consistently with previous reports (Buchon, Broderick, & Lemaitre, 2013; Erkosar & Leulier, 2014), the gut microbiota communities of our flies were dominated by a small number of taxa, in particular by strains of *Acetobacter* and *Lactobacillus*. The ten most abundant OTUs accounted for between 75% and 99.9% of the 16S rRNA gene reads of the community (Figure 1a).

Two different and statistically independent methods of quantifying microbiota abundance consistently indicated a steep increase in abundance with increasing yeast concentration. First, the ratio of 16S rRNA gene reads attributable to gut microbiota to those mapped to the endosymbiont *Wolbachia* ranged from 0.003 on average on the 4% diet, through 0.03 on the 10% diet to 1.1 on the 27% diet (Figure 1b). Second, in an independent assay, we quantified with qPCR the 16S rRNA gene copies attributed the two most abundant bacterial taxa in the gut communities, Acetobacteraceae and Firmicutes, relative to *Drosophila* genome copies. For both taxa, the logarithm of this ratio increased roughly linearly as a function of yeast concentration in the fly diet (Figure 1c,d); the slopes of this increase were nearly identical to each other and to the slope of microbiota to *Wolbachia* ratio (Figure 1b). While both approaches have their limitations (see Methods), the strong agreement between them indicates that the overall abundance of bacteria in the fly guts increased exponentially as a function of yeast content of the food. The 27% yeast diet harbor two orders of magnitude more gut microbes than populations on the 4% diet.

### 3.2 Higher alpha diversity is observed at lower nutrient concentrations

The large variation in the abundance of gut microbes relative to *Wolbachia* resulted in a correspondingly large variation in sampling depth for microbiota reads among populations (1779 to 251,955 reads). The number of OTUs detected per sample ranged from 27 to 48 and did not differ consistently among diets (Data File 1 on Dryad), despite communities on diet richer in yeast being sampled to a much greater depth.

**Figure 2:**
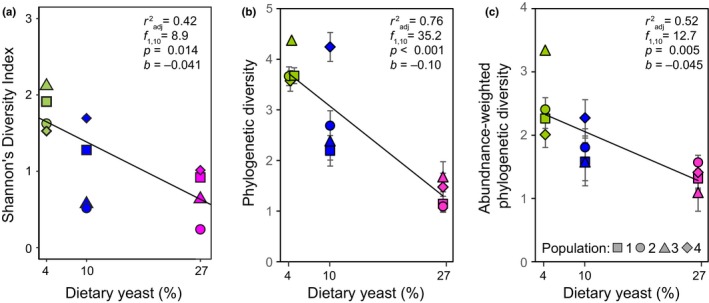
Alpha diversity of gut microbiota depending on the host diet. (a) Shannon's diversity, (b) phylogenetic diversity, and (c) abundance‐weighted phylogenetic diversity. The symbols and error bars (the latter omitted if smaller than the size of the symbol) indicate the means and standard deviations of index estimates calculated from 1000 data sets independently rarefied to the same sampling depth of 1779 reads (see Methods). The fitted lines and the statistics correspond to linear regression on the dietary yeast content; the effect of diet remains significant if diet is treated as a categorical variable (*p *=* *0.014, *p *<* *0.001, *p *=* *0.009 for panel a, b, c, respectively; in all cases, the 27% diet differs from the 4% diet by Tukey's test)

We estimated three measures of alpha diversity: Shannon's Diversity Index, phylogenetic diversity, and abundance‐weighted phylogenetic diversity, on 1000 randomly rarefied subsamples from each sample (see Methods). All three diversity indices declined with increased dietary yeast content, with communities from the 27% diet being on average 45–65% less diverse (depending on the measure) than those from the 4% diet (Figure 2a–c). Microbiota from the 10% diet were intermediate except for those from Population 4, which were as diverse as those from 4% diet. The estimates, particularly those of Shannon's Diversity Index, were fairly robust to variation resulting from random rarefaction (as indicated by the standard deviation bars in Figure 2a–c). Rarefying to 1334 reads (75% of the smallest sample) yielded virtually identical relationships between diet and alpha diversity measures (not shown). Furthermore, the inverse relationship between dietary yeast content and all three measures of alpha diversity remained significant when the analysis was limited to the 20 most abundant OTUs (Appendix: Figure A1).

### 3.3 Differences in community composition upon diet occur mainly at low taxonomic ranks

**Figure 3:**
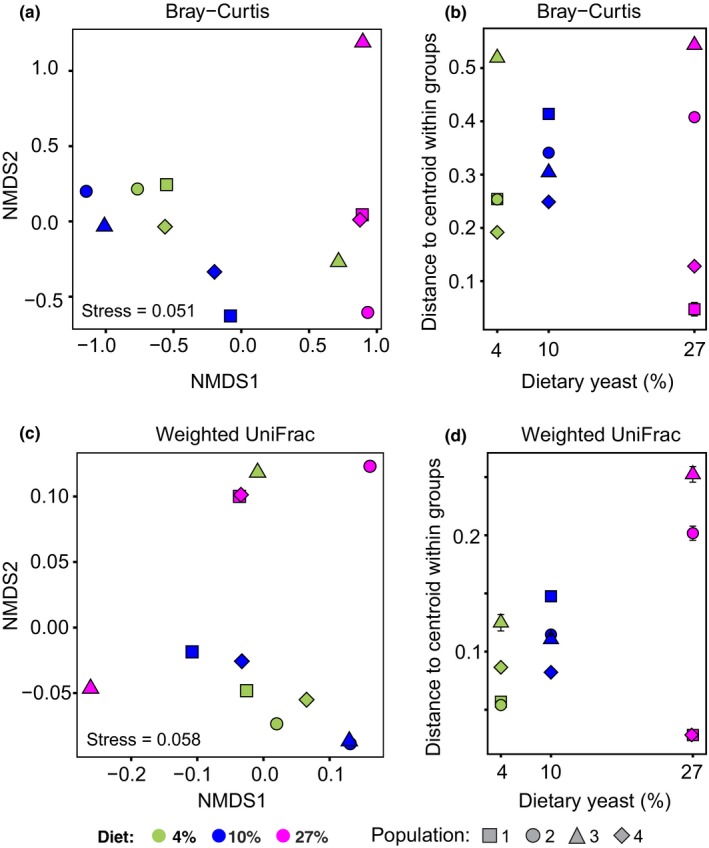
Differences in community composition depending on the host diet. (a, c) Nonmetric multidimensional scaling based on Bray–Curtis dissimilarities (a) and weighted UniFrac distances (c). Both graphs are produced using one rarefied representative community. (b, d) Variability in composition among replicate communities within each diet, based on Bray–Curtis dissimilarities (b) and weighted UniFrac distances (d). Symbols represent mean ± SD Euclidean distances of replicate community compositions to their treatment centroid in the NMDS space, calculated from 100 random rarefactions of each sample

We performed beta‐diversity analyses based on two community distance metrics, Bray–Curtis (Bray & Curtis, 1957) and weighted UniFrac (Lozupone et al., 2007). While both take into account the presence and relative abundance of OTUs, the latter also accounts for the phylogenetic relatedness of detected taxa. As is apparent in Figure 1a, there was considerable variation among replicate communities in the same diet treatment in terms of identity and taxonomic affiliation of the most common OTUs. In particular, while some communities were highly dominated by Acetobacteraceae, others contained a substantial proportion of Firmicutes, and one (that of Population 3 on 27% diet) was strongly dominated by Firmicutes (Figure 1a). As another example of idiosyncratic community composition, community from Population 3 on 4% diet shared its most common OTU with communities 1, 2, and 4 from the 27% diet. As a consequence, nonmetric multidimensional scaling (NMDS) tended to group these populations together (Figure 3a,c). In spite of these idiosyncrasies, permutational multivariate analysis of variance (ADONIS) based on Bray–Curtis distances showed a signal of statistically significant differentiation by diet; however, an analogous analysis based on weighted UniFrac distances did not detect any such differentiation (Table 1).

**Table 1:** Summary of statistics from the permutational multivariate analysis of variance testing for differences in community composition among diets (Adonis) and the permutation tests for homogeneity of multivariate dispersions among diets (Dispersal Permutest). Both tests were performed 100 times following independent rarefaction runs; the numbers are median and range of statistics from those 100 runs. Diet (yeast content) was treated as a continuous variable for Adonis but as a categorical variable for Dispersal Permutest as the latter analysis does not allow for continuous variables

	Adonis			Dispersal Permutest	
	*F*_1,10_	*p*	*R* ^2^	*F*_2,9_	*p*
Bray–Curtis	6.0 [5.4–6.5]	0.002 [0.001–0.005]	0.38 [0.35–0.39]	0.08 [0.04–0.131]	0.92 [0.87–0.97]
Weighted UniFrac	2.0 [1.6–2.2]	0.14 [0.11–0.22]	0.17 [0.14–0.18]	0.45 [0.38–0.89]	0.64 [0.41–0.71]

Taken together, these results suggest that dietary yeast concentration caused a minor shift in the microbiota composition, mostly at the genus or species level. The most apparent aspect of this shift is the replacement of the dominant Acetobacter OTU 549374 in 4% and 10% diets by Acetobacter OTU 273647 on the 27% diet (Figure 1a).

No evidence for heterogeneity of variance among treatment was detected for either Bray–Curtis or weighted UniFrac distances (Table 1). Correspondingly, the distances to treatment centroids were not systematically different among diets (Figure 3b and d, Table 1), nor were the multidimensional estimates of variance in microbiota composition (Table 2). Thus, host diet did not have a detectable effect on the degree of variation among replicate communities exposed to the same diet.

**Table 2:** Multidimensional equivalent of variance in microbiota community composition among replicate host populations: mean squared Euclidean distance of individual communities to the diet treatment centroid in multidimensional space, using both distance matrices (mean and range of values obtained for 100 independent rarefactions)

Distance measure	4% diet	10% diet	27% diet
Bray–Curtis	0.154 [0.149–0.160]	0.184 [0.173–0.194]	0.155 [0.150–0.163]
Weighted UniFrac	0.0084 [0.0053–0.0092]	0.0228 [0.0213–0.0290]	0.0357 [0.0314–0.0436]

## 4 DISCUSSION

We aimed to test the relationship between nutrient supply and microbial abundance and diversity in the gut ecosystem. Therefore, unlike the previous studies, which manipulated the type of nutrients in the diet (e.g., fat‐to‐protein ratio) (David et al., 2014; Mccracken, Simpson, Mackle, & Gaskins, 2001; Ounnas et al., 2016; Perez‐Cobas et al., 2015; Turnbaugh et al., 2008), the diet treatments implemented in this study only differed in the concentration of nutrients and not in their source or type. Nonetheless, the diet had a profound influence on both abundance and alpha diversity of *Drosophila* gut microbiota. The 6.75‐fold increase in dietary yeast content between the 4% and the 27% diet resulted in more than 100‐fold increase in the total microbiota abundance in fly guts (Figure 1b–d). While the quantification of microbiota abundance might be to some degree confounded by differences in the amount of food in the gut, this could not explain a difference of this magnitude (furthermore, the flies on poor diet should eat more and thus contain more rather than less food in their guts). Rather, these results are consistent with the productivity of the bacterial community being higher on richer diets.

In parallel to the increase in abundance, the alpha diversity of the microbial communities, both in terms of OTU diversity and the amount of phylogenetic history contained in the community, decreased with increasing yeast content (Figure 2b,c). The magnitude of this decrease—about 2.5‐fold for Shannon's Index—is considerably greater than diet‐induced changes in alpha diversity (or their absence) reported in gut microbiota studies that manipulated the type of nutrients in the diet (David et al., 2014; Mccracken et al., 2001; Ounnas et al., 2016; Perez‐Cobas et al., 2015; Turnbaugh et al., 2008). In fact, the range of Shannon Index values in our study is nearly as large as that observed across wild‐caught flies from multiple *Drosophila* species feeding on a variety of natural diets ranging from fruits to mushrooms (Chandler et al., 2011). In addition to responding directly to the nutrient supply, the changes in microbiota abundance and diversity may be modulated by physiological responses of the host. Such effects might be expected, given that dietary yeast content has large effects on fly physiology, reproduction, and lifespan (Alic & Partridge, 2011; Gronke, Clarke, Broughton, Andrews, & Partridge, 2010; Piper, Skorupa, & Partridge, 2005), and some of them could affect the mechanisms that modulate the microbiota.

In contrast to most experimental studies on the effect of diet on gut microbial community, which studied short‐term changes in gut microbiota of individual hosts switched to a new diet, we looked at long‐term consequences of dietary regimes applied to whole host populations during multiple generations. Such long‐term microbiota changes may be more profound than short‐term within‐individual changes. The microbes present in the *Drosophila* gut are mostly transient and constantly replenished by ingesting microbes with food (Blum, Fischer, Miles, & Handelsman, 2013; Wong et al., 2015). Thus, the microbiota of flies, and presumably of many other invertebrates, are shared among the members of the host population(s) sharing a food source (Staubach et al., 2013). Furthermore, the microbiota community is not only shaped by processes occurring in the gut, but also—possibly to a greater extent—by the ability of the microbes to persist, grow, and compete in the food substrate. However, the processes occurring in the substrate are also strongly influenced by the insects: The food is colonized by microbes in fly feces and feeding and excretion by flies and larvae change its structure and chemistry. As a consequence, the microbial community in the food substrates mostly consists of *Drosophila*‐associated bacteria and is different from the community colonizing the food in the absence of flies (Martinson, Carpinteyro‐Ponce, Moran, & Markow, 2017; Wong et al., 2015). By maintaining populations on different diets for multiple generations, our study accounted for the effect of nutrient concentration on all these factors.

Furthermore, in natural environments, fly dispersal mediates connectivity of the *Drosophila*‐associated microbial communities among different food patches, resulting in a metacommunity structure. Although in our study the flies were not exchanged among cages, there was ample opportunity for dispersal of microbes: The cages had mesh‐covered openings and were maintained side by side in the same incubator and were handled twice a week to exchange food dishes, presumably resulting in bacteria being transferred between cages on the hands of the researcher. Thus, the *Drosophila* microbiota, both in nature and in our study, likely conform well to Baas–Becking's conjecture about microbial ecology that “everything is everywhere, but the environment selects” (Martiny et al., 2006).

The inverse relationship between nutrient supply and microbial diversity in our study parallels the relationship between ecosystem productivity (as affected by nutrient supply) and diversity often found in plants. However, it contrasts with findings from soil bacteria in agricultural ecosystems (Canfora et al., 2015; Doan et al., 2014; Zhen et al., 2014; Zhong et al., 2010) and in a salt marsh (Kearns et al., 2015), where an increase in nutrient supply—in the form of organic fertilizers—led to long‐term increases in bacterial alpha diversity. The fertilization treatments in those studies likely changed the type of bacterial nutrients in addition to their quantity, and there are obviously many differences between the soil and the gut as bacterial environments. We note, however, that the animal gut is among the most nutrient‐rich bacterial habitats, with bacterial densities several orders of magnitude greater than those found in the soil, in spite of the constant drain on the community through the loss of bacteria with feces (Whitman, Coleman, & Wiebe, 1998). Thus, one possible explanation for this apparent discrepancy is that the relationship between nutrient availability and microbial diversity is hump‐shaped, as predicted by some theoretical arguments (Grime, 1973; Huston, 2014), but the soil and gut microbial communities occupy different ranges of nutrient availability, corresponding, respectively, to the increasing and decreasing portion of the humped curve (see (Smith, 2007) for a similar argument in the context of aquatic microbial diversity patterns).

Shifts in microbiota composition are correlated with changes in host metabolism in mammals (Goodrich et al., 2014), and growth‐promoting effects have been attributed to specific members of the *Drosophila* microbiota (Erkosar, Kolly, Van Der Meer, & Kawecki, 2017; Shin et al., 2011; Storelli et al., 2011). However, whether microbiota diversity in itself affects host fitness remains unclear, although gut microbiota diversity was found to be positively correlated with human metabolic health (Le Chatelier et al., 2013). We only see a minor shift in our data set where the abundance of dominant *Acetobacter* sp. in 4% and 10% diets becomes notably low on 27%. However, our results imply that microbiota abundance declines exponentially with dietary yeast content; yet it is on diets with low yeast content that microbiota become crucial to maintain healthy growth and survival of the *Drosophila* host (Erkosar et al., 2015, 2017; Ridley, Wong, Westmiller, & Douglas, 2012; Shin et al., 2011; Storelli et al., 2011). Thus, the microbiota become scarce under the dietary conditions in which the host particularly needs them. On the other hand, it is unclear to what degree the high abundance of microbiota on rich diets is still a benefit for the host—larval growth and development does not seem to be affected, and gut dysfunction caused by proliferation of microbiota is a major factor in age‐related mortality in *Drosophila*—germ‐free flies live longer (Erkosar & Leulier, 2014). Low yeast concentration (dietary restriction) likewise extends fly lifespan (Partridge, Piper, & Mair, 2005). Given our finding that low dietary yeast content results in much lower abundance of gut microbiota, it is tempting to speculate that the life‐extending effect of low dietary yeast may be partially mediated by reduced microbiota abundance or greater complexity of the bacterial community. Thus, our results support the notion that ecological study of the gut ecosystem will be essential for understanding of the physiological, evolutionary, and health effects of gut microbiota on the host (Shapira, 2016).

## CONFLICT OF INTEREST

None declared.

## AUTHOR CONTRIBUTIONS

BE, AAM, JRvdM, and TJK conceived and designed the study; FZ and UF initiated the experimental fly populations; BE obtained and processed the samples; BE and TJK analyzed the data; EY provided advice on the analysis the data; BE, JRvdM, and TJK wrote the manuscript.

## DATA ACCESSIBILITY

Raw read sequence data have been deposited in the NCBI Sequence Read Archive (BioProject PRJNA477328, sample accessions SAMN09464970‐SAMN09464981). qPCR data and the list of OTUs and read counts are available on Dryad (doi:10.5061/dryad.t0554g0).
