# Supplementary material for: Host diet mediates a negative relationship between abundance and diversity of Drosophila gut microbiota
Source: Ecol Evol. 2018 Aug 29;8(18):9491–502. doi: 10.1002/ece3.4444 (PMC6194258; doi:10.1002/ece3.4444)
Supplement: Supplementary file 1 [file ECE3-8-9491-s001.pdf]

Appendix: Version of figure 2 for data set limited to 20 most abundant OTUs

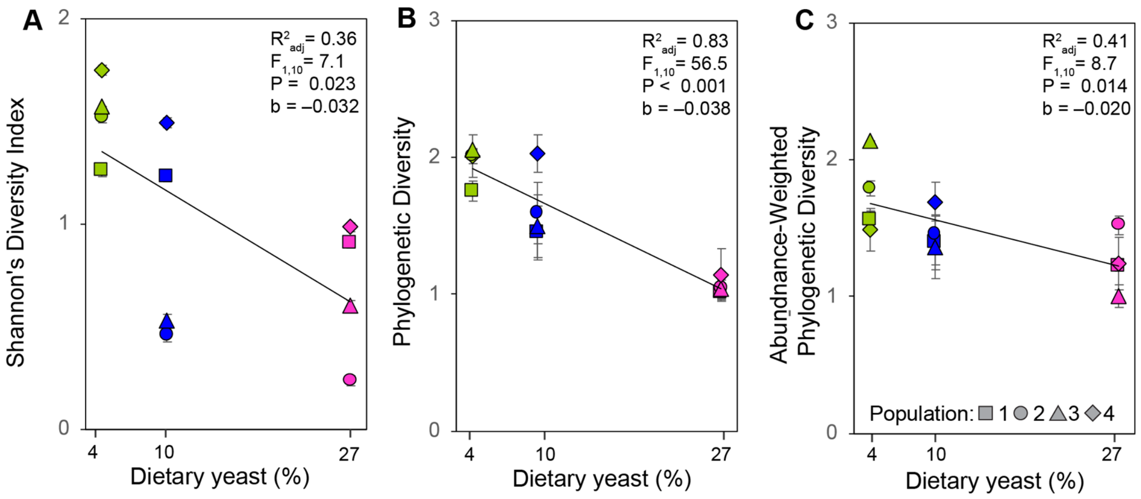

Figure A1. The relationship between host diet and alpha diversity of gut microbiota calculated for a reduced data set limited to 20 most abundant OTUs. (A) Shannon's Diversity, (B) Phylogenetic Diversity, (C) Abundance-Weighted Phylogenetic Diversity. The symbols and error bars (the latter omitted if smaller than the size of the symbol) indicate the means and standard deviations of index estimates calculated from 1000 data sets independently rarefied to the same sampling depth of 1554 reads. The fitted lines and the statistics correspond to linear regression on the dietary yeast content.
